# Efficient and robust RNA-seq process for cultured bacteria and complex community transcriptomes

**DOI:** 10.1186/gb-2012-13-3-r23

**Published:** 2012-03-28

**Authors:** Georgia Giannoukos, Dawn M Ciulla, Katherine Huang, Brian J Haas, Jacques Izard, Joshua Z Levin, Jonathan Livny, Ashlee M Earl, Dirk Gevers, Doyle V Ward, Chad Nusbaum, Bruce W Birren, Andreas Gnirke

**Affiliations:** 1 Genome Sequencing and Analysis Program, The Broad Institute of MIT and Harvard, 320 Charles Street and 301 Binney Street, Cambridge, MA 02141, USA; 2 Department of Molecular Genetics, The Forsyth Institute, 245 First Street, Cambridge, MA 02142, USA; 3 Department of Oral Medicine, Infection and Immunity, Harvard School of Dental Medicine, 188 Longwood Ave, Boston, MA 02115, USA

## Abstract

We have developed a process for transcriptome analysis of bacterial communities that accommodates both intact and fragmented starting RNA and combines efficient rRNA removal with strand-specific RNA-seq. We applied this approach to an RNA mixture derived from three diverse cultured bacterial species and to RNA isolated from clinical stool samples. The resulting expression profiles were highly reproducible, enriched up to 40-fold for non-rRNA transcripts, and correlated well with profiles representing undepleted total RNA.

## Background

Microbial communities are known to play significant roles in human health, development, and disease [1-4], and DNA sequencing is an effective approach to characterize the structure and potential function of these communities. While sequencing of the 16S ribosomal RNA (rRNA) gene has been invaluable for identifying bacterial species and taxa in complex communities [5], shotgun sequencing of metagenomes provides a much richer view of the community by more fully describing the gene content of the community [6,7]. To understand which genes and gene pathways are actually expressed and thus likely functional, however, it is necessary to interrogate the community transcriptome or 'meta-transcriptome'. Accordingly, ultra-high throughput sequencing of transcriptomes, RNA-seq, has rapidly become the method of choice for revealing functional genes and pathways in individual microbes [8-16], as well as in complex environmental communities - for example, from the sea [17,18] and from the human gut [19,20].

Microbial transcriptome sequencing poses significant challenges. Messenger RNA typically constitutes a very small fraction of the total RNA in bacterial cells, as vast amounts of ribosomes and, hence, rRNA are required to meet the cells' demand for protein synthesis. Moreover, the majority of bacterial mRNA is not polyadenylated, as it is in eukaryotes, and can, therefore, not be isolated using oligo-dT selection. Thus, specialized approaches are needed to enrich the desired transcripts for sequence-based characterization.

Numerous rRNA-depletion methods have been developed. These include commercially available kits such as MICROBExpress (Ambion), which uses capture oligonucleotides targeting specific regions of the 16S and 23S rRNAs, and mRNA-ONLY (Epicentre), which utilizes a 5´-monophosphate-dependent exonuclease to degrade processed 5'-phosphorylated RNA molecules such as rRNAs. These kits are widely used, albeit with limited success for meta-transcriptomic purposes. For example, a recent study comparing MICROBExpress and mRNA-ONLY, either alone or in combination, achieved only a modest (1.9- to 5.7-fold) enrichment of bacterial mRNA with less than 25% of aligned sequencing reads representing transcripts other than rRNA [21]. Similarly, subtractive hybridization with non-commercial, sample-specific anti-rRNA probes increased the percentage of non-rRNA reads from phytoplankton RNA no more than about four-fold to slightly less than 50% [22].

Recently, several alternative methods for removal of rRNA have become available: Ribo-Zero, a new hybrid-subtraction kit from Epicentre, promises to remove all species of rRNAs, including the 5S rRNA, from intact and partially degraded total RNA preparations from both Gram-negative and Gram-positive bacteria; the Ovation Prokaryotic RNA-seq System from NuGEN uses a proprietary set of 'not so random' primers to avoid rRNA as template during first and second strand cDNA synthesis similar to the strategy of Armour *et al*. [23]; and degradation of fast re-annealing abundant cDNAs by a duplex-specific nuclease (DSN) [24] has been shown to deplete cDNA representing rRNAs while largely preserving the relative abundance of non-rRNA transcripts in the *Escherichia coli *transcriptome [25].

Our goal was to establish a robust and scalable RNA-seq process applicable to cultured bacteria as well as to complex community transcriptomes. An effective process should a) reduce rRNA sequences to very low levels; b) accurately maintain relative representation of transcript sequences; c) be equally successful for any species; d) work well with RNA of varying quality; and e) be highly reproducible. To this end, we evaluated rRNA depletion methods and chose a protocol that eliminates rRNA reads efficiently and robustly, largely irrespective of the quality of the RNA input sample. We paired this protocol with a strand-specific cDNA synthesis and RNA-seq approach [26] that helps to demarcate the boundaries of adjacent genes and operons that are transcribed from different strands and can distinguish between sense and antisense transcipts of overlapping genes. In addition, for a protocol to be effective in meta-transcriptomic applications, the process a) needs to be effective in diverse species, and b) does not require high rRNA integrity, which is often difficult to obtain with clinical samples. Thus, as a technical validation we demonstrated the effectiveness of our optimized process with RNA extracted from human stool samples.

## Results

### Evaluation of rRNA depletion methods

To provide a benchmark for method evaluation, we prepared RNA from three well characterized organisms (*Prochlorococcus marinus*, *Escherichia coli*, and *Rhodobacter sphaeroides*) that cover a wide range of base compositions (30%, 51%, and 69% genome GC content, respectively). We prepared total RNA from each organism and used these samples separately or as a 'PER' pool (mixed 1:1:1 by mass) of input material.

We compared five methods for removing rRNA: three commercially available rRNA depletion kits (MICROBExpress, mRNA-ONLY, and Ribo-Zero), a commercial kit for 'not-so-random' primed cDNA synthesis of non-rRNA templates (Ovation Prokaryotic RNA-Seq System), and a protocol for removing highly abundant cDNAs by low-c_0_t re-annealing and light normalization of cDNA libraries using DSN [25].

We constructed RNA-seq libraries from total (undepleted) RNA and rRNA-depleted samples using each of the methods, sequenced them using the Illumina platform, mapped the reads to the three reference genome sequences and separately counted reads that aligned to rRNA and to the coding DNA sequence (CDS) of annotated genes (see Materials and methods). Read counts for protein-coding genes were normalized for CDS lengths and for the total number of sequencing reads by calculating RPKM (reads per kb per million mappable reads) values [27] for each expressed gene. Bias and dropouts in mRNA expression profiles introduced during the depletion process were assessed by analyzing the linear correlation of gene expression values before and after rRNA depletion (scatter plots available in Figure S1 in Additional file 1).

**Figure 1:**
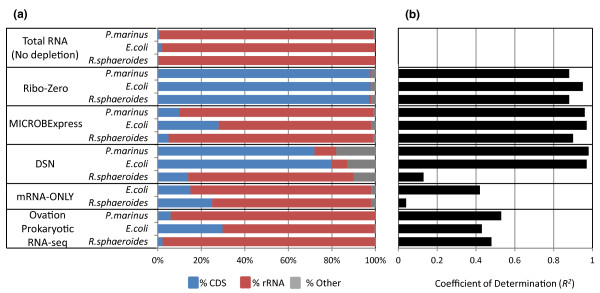
**Performance evaluation of five rRNA depletion methods**. **(a) **Shown is the distribution of RNA-seq reads aligning to protein-coding sequences (CDS; blue), rRNA (red), and other regions (tRNA, non-coding RNA, small RNA, and intergenic regions; gray) for undepleted total RNA (top) and five rRNA depletion protocols. **(b) **The lengths of the black bars represent the coefficient of determination (*R^2^*) for RPKM values before and after rRNA depletion using different rRNA-depletion methods. Ribo-Zero, normalization using duplex-specific nuclease (DSN) and Ovation were tested on a 1:1:1 pool (by mass) of total RNA prepared from *P. marinus*, *E. coli*, and *R. sphaeroides*. MICROBExpress and mRNA-ONLY were performed on individual RNA preparations without pooling.

Without depletion, for all three organisms almost all mapped reads (>98%) aligned to rRNA (Figure 1a top, red bars). Each of the methods tested resulted in rRNA depletion, but to varying extents. Overall, Ribo-Zero performed the best, dramatically diminishing the percentage of rRNA reads for all three species to less than 1%. Conversely, CDS reads increased from ≤2% to 97 to 98% of total reads (blue bars). Importantly, RPKM values of expressed genes in the depleted library were strongly correlated to those in the original total RNA control (Figure 1b top; *R^2 ^*= 0.88, 0.95, and 0.88 for *P. marinus*, *E. coli*, and *R. sphaeroides*, respectively), indicating little, if any, systematic skewing caused by the rRNA-subtraction procedure.

Light normalization (DSN) reduced the proportion of rRNA reads appreciably for *P. marinus *and *E. coli *(from 98% to 10% and 7%, respectively); however, it did not perform well for the GC-rich *R. sphaeroides *transcriptome (99.5% rRNA reads before and 76% after depletion; *R^2 ^*= 0.13). A detailed analysis of the CDSs for *R. sphaeroides *pointed to a high GC content as a major adverse factor (Figure S2 in Additional file 1). The normalization protocol enriched a small fraction of CDSs very well (>10-fold). This group (9% of all CDSs in *R. sphaeroides*) had a moderate mean GC content of 57%. The majority (83%) of transcripts were poorly enriched (<2-fold) or even depleted (68% GC on average within this group). It is possible that the high-GC fraction anneals faster or forms hairpins during the re-association reaction and, thus, becomes a substrate for degradation by DSN, thereby making it more difficult to enrich GC-rich CDSs relative to *R. sphaeroides *rRNA, which is only about 56% GC.

MICROBExpress maintained a strong correlation between the total and rRNA-depleted PER pool (*R^2 ^*= 0.96, 0.97, 0.90 for *P. marinus*, *E. coli*, and *R. sphaeroides*, respectively); however, it was not effective in removing *P. marinus *and *R. sphaeroides *rRNAs (89% and 94% residual rRNA reads, respectively) and was only slightly better for *E. coli *(70% rRNA reads remaining despite rRNA depletion). Neither mRNA-ONLY nor Ovation reduced the fraction of rRNA reads below 70%. Moreover, neither protocol generated CDS-expression profiles that correlated well with the undepleted total RNA control (*R^2 ^*values ranging from 0.04 to 0.53).

**Figure 2:**
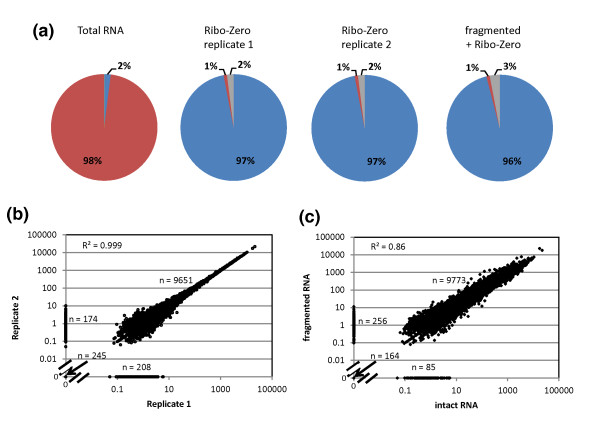
**Depletion of rRNA in a mixture of total RNAs from *E. coli*, *R. sphaeroides *and *P. marinus *with Ribo-Zero is reproducible and works well with fragmented total RNA**. **(a) **The pie charts represent the mapped read distributions of protein-coding genes (CDS; blue), rRNA (red), and other reads (tRNA, non-coding RNA, small RNA and intergenic regions; gray) for undepleted total RNA, two technical replicates of Ribo-Zero treatment of intact total RNA and for Ribo-Zero treatment of fragmented total RNA. **(b,c) **Double-log scatter plots of RPKM values and the coefficient of determination (*R^2^*) for the technical Ribo-Zero replicates (b) and for Ribo-Zero treatment of fragmented versus intact total RNA (c). Points on the axes represent CDSs with zero coverage in one of the two samples. The number of data points in the diagonal cloud and on the axes is indicated. The total number of annotated CDSs in the three bacterial genomes is 10,278.

In our hands, Ribo-Zero all but eliminated rRNA reads, thereby enriching CDS reads approximately 40-fold without skewing the expression profile of protein-coding genes compared to the original, total PER RNA pool. Further, we found removal of rRNA with Ribo-Zero to be highly reproducible. In each of two replicates, starting with the same PER total RNA pool, only 1% of the resulting reads mapped to rRNA (Figure 2a). RPKM measurements ranged over five orders of magnitude from <10^-1 ^to >10^4^. The correlation between technical replicates was excellent (*R^2 ^*= 0.999). Cases of CDSs being present in only one or the other of two experiments were confined to rare transcripts with RPKM values <10 (Figure 2b).

To evaluate the effectiveness of the Ribo-Zero method on low quality RNA samples, we created a simulated low quality sample by fragmenting total PER RNA artificially such that the mode of the fragment size distribution was approximately 300 bases (Figure S3 in Additional file 1). The fragmented sample was Ribo-Zero treated and processed in parallel with the two replicates of the standard process as previously described. Fragmenting undepleted total RNA led to a slight increase in the percentage of reads aligning to tRNA and other non-coding or unannotated regions (Figure 2a; gray) but not to an increase in the percentage of rRNA reads (1% for both intact and fragmented input RNA; Figure 2a, red). Importantly, >95% of the reads from the artificially fragmented RNA mapped to protein-coding sequences (Figure 2a, blue), and the expression profiles obtained from intact and artificially fragmented total RNA were highly correlated (Figure 2c; *R^2 ^*= 0.86).

The fraction of CDSs detectable at different RPKM thresholds from 0.1 to 100 with or without rRNA depletion in all three transcriptomes are shown in Figure S4 in Additional file 1. Removal of rRNAs from both intact and fragmented RNA greatly enhanced the detection sensitivity for protein-coding transcripts. The percentage of CDSs with RPKM values ≥1 increased up to two-fold following Ribo-Zero treatment (from 47 to 63% to 87 to 94% for intact RNA and 82 to 88% for fragmented RNA). Input RNA quality also affected the sensitivity of detection. The sensitivity for the lower abundance transcripts was slightly lower for the fragmented compared to the intact transcripts.

### Strand specificity

**Figure 3:**
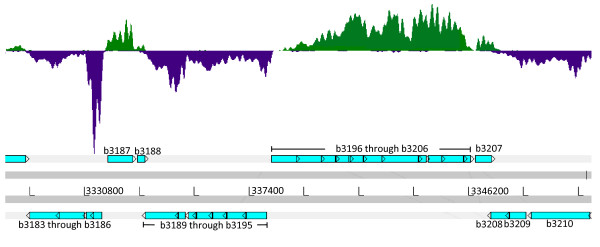
**Strand specificity of RNA-seq reads**. Shown is a 17-kb window of the *E. coli *genome viewed with the Artemis browser [28]. The mapped reads aligning to the top strand (green) or bottom stand (purple) consistent with the direction of the annotated genes as represented by the blue boxes with arrows and corresponding gene ID numbers and operons below (for example, genes b3196 through b3206).

To add the capability to generate strand-specific reads that distinguish between RNA transcribed from the two strands of DNA, we adopted dUTP marking and degradation of second strand cDNA [26]. An Artemis genome browser image [28] illustrating the coverage with reads aligned to the top strand (green) or the bottom strand (purple) within a typical segment of the *E. coli *reference genome is shown in Figure 3. The vast majority of read alignments was consistent with the known direction of transcription of annotated genes and operons in this interval.

To measure the strand specificity of our method, we calculated separate sense and antisense read densities for all members of a curated list of protein-coding genes. The median strand specificity for genes that were expressed in the top 10% expressed members was 99.8% (*P. marinus*), 99.8% (*E. coli*) and 99.9% (*R. sphaeroides*). Results for other quartiles are presented in Figure S5 in Additional file 1. A technical replicate generated from the same starting PER RNA pool gave essentially the same results.

### Application to stool samples

To test our protocol on real clinical samples, we extracted and sequenced DNA and RNA from two human stool samples. RNA from stool A had a high RNA integrity value (RIN = 9) whereas RNA from stool B was partially degraded (RIN = 7).

To determine the bacterial composition of the two stool samples, we aligned genomic DNA-seq reads to 649 bacterial reference genomes (Additional file 2; see Materials and methods) and an Archaeon reference (*Methanobrevibacter smithii *ATCC 35061). Subsequent alignment of the RNA-seq reads showed that the majority of the reads mapped to the 19 most abundant genomes in the stool samples (Additional file 3) and were used for gene expression analyses. The distribution of DNA and RNA-seq reads among these 19 species is shown in Figure S6 in Additional file 1. *Prevotella copri *was the most prevalent species, represented by almost half of the genomic reads from these 19 species in both stool samples. It also dominated the meta-transcriptomic data, with 87% and 70% of total RNA-seq reads for the top 19 species in stools A and B, respectively.

**Figure 4:**
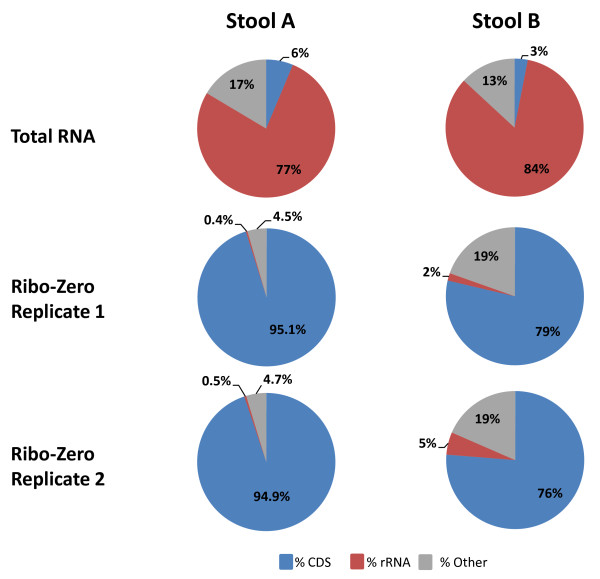
**Bacterial rRNA depletion and CDS enrichment in RNA extracted from two human stool samples**. Shown is the distribution of sequencing reads aligning to annotated protein coding genes (CDS; blue), rRNA (red) and other features (tRNA, intergenic regions and contigs with no annotations; gray) of 19 reference genomes representing the most abundant bacterial species in stools A and B.

The percentage of rRNA reads in total RNA (77% for stool A and 84% for stool B; Figure 4) was lower than for the composite PER total RNA pool (>98% rRNA; Figure 2a), possibly reflecting a bacterial community with slower growth rates in a natural environment compared to cells growing exponentially in liquid monoculture in the lab.

**Figure 5:**
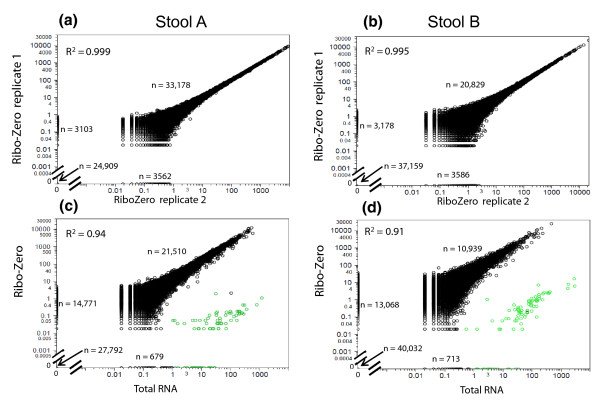
**Ribo-Zero depleted gene-expression profiles from intact and partially degraded human stool samples: technical reproducibility and correlation with total RNA-seq data**. **(a-d) **Shown are double-log scatter plots and the coefficient of determination (*R^2^*) comparing RPKM values for 64,752 annotated CDSs (points in black) in 19 bacterial genomes between technical Ribo-Zero replicates (a,b) as well as with and without Ribo-Zero depletion (c,d). RNA extracted from stool A was largely intact (RIN = 9). RNA from stool B was partially degraded (RIN = 7). Input amount was 5 µg total RNA. Points on the axes represent CDSs without coverage in one of the two samples. The number of data points on the diagonal and on the axes is indicated. Points in green represent the unannotated rRNAs.

Ribo-Zero treatment, carried out in replicates, reduced the rRNA reads to <1% for stool A and to 2 to 5% in the partially degraded stool B sample (Figure 4). Conversely, the CDS reads increased to approximately 95% in stool A and to 76 to 79% in stool B replicates. Technical reproducibility of meta-transcriptomic expression profiles was excellent for both samples (*R^2 ^*= 0.999 and 0.995) without dropouts over four orders of magnitude (Figure 5a,b). CDSs covered in only one of two duplicate experiments had RPKM values <1 in stool A and <3 in stool B. None of the reads mapped to *M. smithii*; therefore, we were unable to evaluate the perfomance of Ribo-Zero on Archaea.

While rRNA depletion did not skew the expression profiles of the vast majority of protein-coding genes compared to RPKM values in undepleted total RNA (*R^2 ^*= 0.94 and 0.91 for stools A and B, respectively; Figure 5c,d), a small number of transcripts (highlighted in green) fell clearly below the diagonal of the scatter plot or dropped out completely. On closer examination, this subset (n = 163, total for both samples) comprised unannotated rRNAs and putative CDSs that overlapped with rRNA genes, thereby explaining their depletion along with *bona fide *rRNAs (Additional file 4) and, thus, they were excluded from the correlation.

Ribo-Zero performed effectively on both Gram-negative and Gram-positive bacteria. We calculated species-specific gene-expression data for two Gram-negative bacteria, *Prevotella copri*, the most abundant species in both stool samples, and *Bacteroides vulgatus*, ranked fifth and fourth by the number of genomic reads and second and third by CDS RNA-seq reads in stools A and B, respectively, as well as for the Gram-positive *Eubacterium rectale*, ranked second by genomic reads and third by CDS RNA-seq in stool A. Technical replicates using 5 µg of input RNA had *R^2 ^*values of 0.99 or higher for all three bacterial transcriptomes in either stool sample (Figures S7 and S8 in Additional file 1).

We evaluated the performance of this method with less than 5 µg of input RNA. Lowering the input amount of the RIN 9 RNA sample from 5 µg down to 50 ng and 5 ng led to a partial loss and almost a complete loss of the mapped reads to the top 19 species (from 96% to 42% and 0.4%, respectively) and a concomitant increase of mapped reads to *E. coli *(up to 95%; Additional file 3). It is, therefore, not advisable to use the Ribo-Zero kit with RNA quantities below the manufacturer's recommendations. Approximately 0.5 to 1% of the RNA is recovered following Ribo-Zero treatment; thus, further development may be necessary for this method with lower amounts of RNA. Typical amounts of RNA or cDNA recovered at each step of the process are given in Additional file 5.

## Discussion

We developed a robust RNA-seq process that is applicable both for bacteria grown in culture and for complex bacterial communities from clinical samples, and in so doing have overcome the major challenge to microbial meta-transcriptomics: a universal rRNA depletion method that does not require high quality RNA. First, we compared five different methods for rRNA depletion and identified one protocol, Ribo-Zero, that works well for both intact and fragmented total RNA representing a wide range of base compositions. Second, we combined this method with a strand-specific RNA-seq approach [26] to distinguish between sense and antisense transcripts from a given locus. Third, we tested the process with high-quality and partially degraded RNA extracted from clinical stool samples.

Prior to our evaluation, MICROBExpress was considered the standard for microbial meta-transcriptomics applications despite limitations that included results being strongly affected by both community composition and RNA integrity, and that the method did not produce RNA-seq data sets containing less than 75% rRNA reads [21]. By contrast, in this study we were able to generate RNA-seq data that were predominantly protein-coding sequence. Following Ribo-Zero treatment, more than 95% of aligned reads from high-quality RNA (RIN ≥ 9) aligned to the annotated CDSs. This strong enrichment greatly enhances the ability to detect and quantify rare transcripts, yet with little systematic skewing of expression profiles based on expression level or GC content. Thus, the protocol affords a more comprehensive view of gene expression within complex microbial communities.

To increase the power of the method, we incorporated dUTP marking and degradation of second strand cDNA to generate RNA-seq libraries that preserve the orientation of the expressed transcripts [26]. This protocol is easy to use, compatible with paired-end Illumina sequencing and produces highly strand-specific and quantitative gene-expression data for eukaryotes [29]. Strand specificity adds significant value to RNA-seq data for bacteria. It enables high-throughput studies of regulatory antisense transcripts and facilitates annotation of genes and operons, and helps assign expression values to the correct transcription unit.

Despite our technical advances, the RNA-seq process is still limited by RNA input requirements. For cultured bacterial cells or microbial communities from stools, sufficient RNA starting material is usually obtainable, but input RNA can be limiting with other human or environmental samples. Based on our results, 5 µg of total RNA give excellent results. We have shown that libraries can be made effectively with as little as 5 ng input RNA; however, the Ribo-Zero kit does not perform effectively below microgram amounts of RNA and should be avoided. Several meta-transcriptomics studies resorted to polyadenylation and RNA amplification [17] or multiple-displacement amplification of cDNA by phi29 DNA polymerase [18] for submicrogram amounts of RNA or cDNA, respectively. Both amplifications select against rRNA. Since these studies were performed on unknown, complex communities, it remains unclear what biases were associated with these methods. For samples with extremely small amounts of starting material, skipping the rRNA depletion step to preserve the complexity of the sample for cDNA library construction and sequencing much more deeply may ultimately be the only practical approach.

Currently, large-scale metagenomics projects are rapidly cataloging microbial genes from habitats of the human body, including the gut (The European MetaHIT consortium [7]), nasal cavities, oral cavity, urogenital tract and the skin (Human Microbiome Project [6]), while the more ambitious Earth Microbiome Project [30] aims to catalog all the world's microbes. Having a catalog of genes and gene pathways is a powerful tool, but understanding function will require a scalable meta-transcriptomics approach. To our knowledge, our bacterial RNA-seq process is the only method to date that has carefully evaluated the biases associated with commercially available rRNA depletion kits on real clinical stool samples. This method can be used for clinical as well as environmental samples.

We note that nearly all steps in our process are amenable to automation, including nucleic acid extraction, cDNA synthesis and construction of Illumina sequencing libraries, and can thus be easily scaled to process large numbers of samples. With the exception of the rRNA depletion step with Ribo-Zero, all RNA fragmentation and enzymatic steps are cleaned up using automation-friendly paramagnetic beads; therefore, cDNA synthesis can be automated, as has been demonstrated with a similar protocol [31]. Our Illumina sequencing library construction is based on an automated protocol that uses a single well for multiple subsequent reaction steps to minimize sample loss and streamline sample processing [32]. Finally, depending on the complexity of the community, samples can be indexed and pooled for sequencing.

## Conclusion

We have devised a robust and scalable process for bacterial RNA-seq that combines an efficient rRNA-removal protocol, automation-friendly cDNA-library construction, and maintenance of strand information. This process represents a significant improvement over previous methods and can be applied to profile gene expression in both simple and complex, naturally occurring bacterial communities.

## Materials and methods

### Bacterial culture

*E. coli MG1655 *and *R. sphaeroides 2.4.1 *(ATCC, Manassas, VA, USA) were grown in 250 ml LB broth with shaking at 37°C and 30°C, respectively, to an OD_600 _of about 0.5. Each culture was divided into 50 ml aliquots. Cells were harvested by centrifugation at 4,000 × g for 10 minutes at room temperature. Pellets were resuspended in 25 ml of RNAlater (Ambion, Carlsbad, CA, USA). The tubes were agitated on a rotator at 4°C overnight, centrifuged at 4,000 × g for 10 minutes, placed in an ethanol/dry ice bath to flash freeze the pellet and stored at -80°C.

Fresh *P*. *marinus *(MED4) cultures were a kind gift from Sallie Chisholm (MIT, Cambridge, MA, USA). Cells were grown in 8 L of PRO99 Sargasso sea water medium [33] in a 24°C incubator with a simulated daily light/dark cycle, 13:11 light:dark cycle at 60 μmol Q m-2 s-1 [34-37]. Cells were harvested at mid-log phase based on the fluorometric detection of bulk chlorophyll autofluorescence using a Turner Designs 10-AU Fluorometer. The culture was divided into 250 ml tubes and centrifuged at 15,000 × g for 10 minutes. After removal of the supernatant, RNAlater (50 ml) was added to each tube and the pellet resuspended. Half the cells in RNAlater (25 ml) were transferred into two 50 ml tubes and placed on a rotator overnight at 4°C. The tubes were centrifuged at 23,000 × g for 30 minutes, placed in an ethanol/dry ice bath to flash freeze the pellet and stored at -80°C.

### RNA and DNA extraction

Bacterial cell pellets stored at -80°C in RNAlater (25 ml) were thawed on ice, resuspended and re-pelleted in 1 ml aliquots for 10 minutes at 4,000 × g in a microcentrifuge. The supernatant was removed and 200 μl bacterial lysis buffer (30 mM Tris·HCl, pH 8.0, 1 mM EDTA plus 15 mg/ml lysozyme (Sigma, St Louis, MO, USA) and 15 μl proteinase K (20mg/ml; QIAGEN, Valencia, CA, USA) were added to each tube. Samples were incubated at room temperature for 10 minutes, and vortexed for 10 s before and every 2 minutes during the incubation. QIAGEN RLT Plus buffer (750 μl) supplemented with 1 % v/v beta-mercaptoethanol (Sigma) was added to each tube and vortexed briefly to mix.

Two stool samples were collected 7 months apart from a single human donor (approved collection protocol by the Forsyth Institute Institutional Review Board, Assurance FWA00000398). Approximately 200 mg of stool were placed in approximately 2 ml RNAlater buffer, briefly mixed to disperse matter, and stored at room temperature during transport to the lab. The first sample (stool A) was extracted within 5 h of collection. The second sample (stool B) was stored at 4°C upon arrival for 24 h and then frozen and stored at -20°C for approximately 5 days until extracted. Prior to extraction, samples were vortexed briefly and centrifuged for 10 minutes at approximately 16,000 × g in a microcentrifuge at room temperature. Bacterial lysis buffer (100 μl) plus 10 μl proteinase K (20mg/ml) was added to half the stool sample (approximately 100 mg). Samples were incubated at room temperature for 10 minutes and vortexed for 10 s before and every 2 minutes during the incubation. QIAGEN RLT Plus buffer (1.2 ml) containing 1 % v/v beta-mercaptoethanol was added to each tube and vortexed briefly to mix. Samples were transferred into 2 ml sterile bead beating tubes (BioSpec Products Inc., Bartlesville, OK, USA) filled with 1 ml of 0. 1 mm glass beads (BioSpec Products), and placed in a bead beater (Mini Bead beater-8; BioSpec Products) for 3 minutes on 'homogenize' setting.

The lysed bacterial and stool samples were homogenized using QIAshredder spin columns (QIAGEN) and added to the AllPrep DNA spin columns for RNA and DNA isolation following the manufacturer's protocol. RNA integrity values (RIN values) [38] were determined by running 1 μl aliquots on a Bioanalyzer 2100 (Agilent, Santa Clara, CA, USA). The RNA and DNA were stored in 5 to 20 μg aliquots at -80°C and -20°C, respectively.

### Ribosomal RNA depletion methods performed at RNA level

#### Ribo-Zero

An early access version of Meta-Bacteria Ribo-Zero rRNA removal kit (Epicentre, Madison, WI, USA) was used according to the manufacturer's instructions. RNA input amounts determined the amount of Ribo-Zero rRNA removal solution to add (10 µl rRNA removal solution for 2.5 to 5 µg or 8 µl for <2.5 µg total RNA per reaction). Samples in Ribo-Zero rRNA removal solution were incubated at 68°C for 10 minutes followed by a 15 minute incubation at room temperature. To remove the hybridized rRNA molecules from the mRNA, the RNA/rRNA solution reactions were incubated with the prepared microsphere beads, mixed well and placed at room temperature for 10 minutes (mixing every few minutes), then at 50°C for 10 minutes. The mRNAs were separated from the microspheres bound with rRNAs by a filter column provided in the kit. The final purification of eluted mRNA was performed using Agencourt RNAClean XP beads (2× the volume per mRNA volume; Beckman Coulter Genomics, Danvers, MA, USA).

#### MICROBExpress

MICROBExpress (Ambion/Applied Biosystems, Austin, TX, USA) was used according to the manufacturer's specifications. Briefly, total RNA (5 to 10 μg) was combined with binding buffer (200 μl) and capture oligonucleotide mix (4 μl). The RNA mix was heated to 70°C for 10 minutes then incubated at 37°C for 15 minutes to hybridize the capture oligos. The RNA/capture oligo mix was equilibrated with oligomag beads (50 μl, pre-warmed to 37°C) and incubated at 37°C for 15 minutes. Tubes were placed on a magnet to separate the supernatant containing the enriched mRNA from the oligomag beads. The enriched mRNA was purified and concentrated by ethanol precipitation according to the manual with precipitation at -80°C for 1 h.

#### mRNA-ONLY Prokaryotic mRNA Isolation Kit

Enzymatic reactions using the mRNA-ONLY Prokaryotic mRNA Isolation Kit (Epicentre) were performed according to the manufacturer's specifications. Briefly, total RNA (5 to 10 μg) was combined with 2 μl mRNA-ONLY 10× reaction buffer, 0.5 μl ScriptGuard RNase Inhibitor, 1 μl Terminator Exonuclease (1 U) and nuclease free water in a final volume of 20 μl and incubated at 30°C for 60 minutes. Reactions were terminated with the addition of 1 μl of mRNA-ONLY stop solution (100 mM EDTA). Agencourt RNAClean XP beads (0.8× of the reaction volume) were used to purify the reaction according to the manual.

### DNase treatment

The TURBO DNA-free kit (Ambion) was used for the DNase treatment. Total RNA or rRNA depleted RNA (following MICROBExpress, mRNA-ONLY, or Ribo-Zero treatment) was treated using a rigorous protocol that includes a second addition of DNase (2 to 4 units) and incubation at 37°C for 30 minutes according to the manufacturer's specifications. Reactions were terminated with the addition of the DNase inactivation reagent (0.2× the reaction volume) and purified using Agencourt RNAClean XP beads (0.8× of the reaction volume) according to the kit instructions. The presence of DNA contamination was assessed by PCR with 16S-specific primers. Each reaction included 1× AccuPrime PCR buffer II (10×), 0.75 U of AccuPrime Taq High Fidelity polymerase (5 U/μl; Invitrogen) and 200 nM of each primer (357F: 5'- CCTACGGGAGGCAGCAG -3' and 926R: 5'- CCGTCAATTCMTTTRAGT -3'). The DNase treated RNA (2 μl) was added to each reaction in a final reaction volume of 20 μl. Each reaction was run in parallel with a positive (*E. coli *DNA) and negative (nuclease free water) amplification control. The plates were sealed, centrifuged briefly, and placed in the thermal cycler (ABI 9700, Applied Biosystems, Foster City, CA, USA) with the following cycling conditions: 95°C for 2 minutes, 30 cycles of 95°C for 20 s, 50°C for 30 s, 72°C for 5 minutes. If an amplification product of approximately 600 bp was observed, the RNA was treated again with DNase.

### RNA fragmentation

RNA fragmentation reactions were performed using fragmentation buffer (5×; GeneChip Sample Cleanup Module; Affymetrix, Santa Clara, CA) in a final concentration of 1× per reaction. A maximum of 5 μg of RNA was added to each 10 μl fragmentation reaction, incubated at 80°C for 4 minutes on a thermal cycler, and placed on ice. Agencourt RNAClean XP beads (2.0× of the reaction volume) were used to purify the reactions according to the manual. An RNA fragment size distribution with a mode of approximately 300 bases was achieved with these conditions.

### cDNA synthesis

cDNA synthesis was performed as previously described [29]. Total or rRNA depleted RNA was combined with 3 μg of the random primers (Invitrogen; 3 μg/μl) in a final volume of 11 μl. The reaction was incubated at 70°C for 10 minutes and placed immediately on ice. The remaining reagents were added to the reaction in a final volume of 20 μl: 1× of first strand buffer (10×; Invitrogen), 10 mM of DTT (0.1 M; Invitrogen), 0.5 mM of dNTP mix (10 mM; Invitrogen), 20 U of SUPERase-In (20 U/μl; Ambion) and 200 U of SuperScript III (200 U/μl; Invitrogen). The first strand reaction was incubated at 25°C for 10 minutes followed by 55°C for 60 minutes and then placed on ice. The second strand was synthesized by adding 1× of second strand buffer (5×; Invitrogen), 0.2 mM of dNTPs (10 mM; Invitrogen), 40 U of *E. coli *DNA polymerase I (10 U/μl; NEB, Ipswich, MA, USA), 10 U of *E. coli *DNA ligase (10 U/μl; NEB), 5 U of RNase H (5 U/μl; Invitrogen) to the first strand reaction (150 μl total volume). After 2 h at 16°C, the reaction was stopped by adding 10 μl of 0.5 M EDTA and purified using MinElute PCR Clean up columns (QIAGEN) according to the manufacturer's instructions.

Strand-specific cDNAs were made by dUTP marking and degradation of second strand cDNA [26] using a modification of the protocol by Levin *et al*. [29].. Total or rRNA depleted RNA was combined with 3 μg of the random primers (Invitrogen; 3 μg/μl) in a final volume of 7 μl, incubated at 70°C for 10 minutes and immediately placed on ice. The remaining reagents were added to the first strand synthesis reaction for a total volume of 20 μl: 1× of first strand buffer (5×; Invitrogen), 10 mM of DTT (0.1 M; Invitrogen), 0.5 mM of dNTP mix (10 mM; Invitrogen), 4 μg of Actinomycin D (USB, Cleveland, OH, USA), 20 U of SUPERase-In (20 U/μl; Ambion) and 200 U of SuperScript III (200 U/μl; Invitrogen). The first strand reaction was incubated at 25°C for 10 minutes followed by 55°C for 60 minutes and then placed on ice. The first strand reaction was purified with Agencourt RNAClean XP beads (2.0× of the reaction volume) to remove the Actinomycin D and dNTPs. The second strand synthesis reaction included 1× first strand buffer (5×), 1 mM of DTT (0.1 M; Invitrogen), 260 nM dNTPs (10 mM deoxynucleotide mix containing dUTP instead of dTTP; Roche Applied Science, Indianapolis, IN, USA), 1× second strand buffer (5×), 40 U of *E. coli *DNA polymerase I (10 U/μl; NEB), 10 U of *E. coli *DNA ligase (10 U/μl; NEB) and 5 U of RNase H (5 U/μl; Invitrogen) in a final volume of 150 μl. The second strand reaction was incubated at 16°C for 2 h. The reaction was stopped by adding 10 μl of 0.5 M EDTA and purified using MinElute PCR clean up columns (QIAGEN) according to the manufacturer's instructions or Agencourt AMPure XP beads (2.0× of the reaction volume).

### Selective cDNA synthesis: the Ovation Prokaryotic RNA-Seq System

The Ovation Prokaryotic RNA-Seq System (NuGEN Technologies, Inc., San Carlos, CA, USA) was used as follows. Intact RNA was DNase treated as described above and synthesized into cDNA according to the manufacturer's protocol. Briefly, the first strand primer was mixed with the intact RNA (500 ng/reaction), incubated at 65°C for 5 minutes, and placed on ice. First strand buffer and enzyme mix were added to each tube, mixed well, and incubated at 40°C for 30 minutes, 85°C for 5 minutes and a 4°C hold. Reaction Enhancement Enzyme mix was added to each tube and incubated at 37°C for 15 minutes with a 4°C hold. Second strand primer mix was added to the first strand reaction and incubated at 65°C for 5 minutes with a 4°C hold. The second strand master mix was added to each tube, mixed well and incubated at 25°C for 60 minutes with a 4°C hold. The cDNA was purified using a MinElute column (QIAGEN) and eluted in 1× low TE (10 mM Tris, 0.1 mM EDTA, pH 8.0). The cDNA was sheared using the Covaris S2 adaptive focused acoustics instrument (Covaris, Woburn, MA, USA) with the following conditions: duty cycle 5%, intensity 10, cycles/burst 200, time 6 minutes. The sheared products were purified and concentrated with Agencourt AMPure XP beads (2× the reaction volume).

### Illumina sequencing libraries

Libraries for Illumina sequencing [39] were made with NEB reagent kits and paired-end adapters using modified PCR amplification conditions to minimize base-composition bias [40]. To simplify and streamline the process, especially for low input libraries, we transitioned to the 1 tube 'with bead' method [32] in which all the steps (end repair, A-base addition and adaptor ligation ± indexing) were carried out in a single tube. Following adaptor ligation, the purified products were size selected on a gel (approximately 300 to 450 bp). cDNAs created with the second strand dUTP approach were treated with 1 U Uracil-Specific Excision Reagent enzyme mix (USER; NEB) at 37°C for 15 minutes followed by 95°C heat inactivation for 5 minutes. Samples were enriched with Illumina PE1.0 and PE2.0 primers (1 μM each), 1× of AccuPrime PCR buffer I (10×), 0.5 U of AccuPrime Taq High Fidelity polymerase (5 U/μl; Invitrogen) in a final volume of 25 μl. Enriched reactions were purified using Agencourt AMPure XP beads (0.8× the reaction volume).

### Low C_0_t normalization of cDNA libraries using duplex-specific nuclease

The enrichment protocol following adaptor ligation was modified using 0.5 μM of each truncated paired end adaptor primer (SBS3_8 nt_F: 5'- TACACGACGCTCTTCCGATCT-3' and SBS8_7nt_R: 5' - CTGAACCGCTCTTCCGATCT-3'), 1× of AccuPrime PCR buffer I (10×), 0.5 U of AccuPrime Taq High Fidelity polymerase (5 U/μl; Invitrogen) in a final volume of 25 μl. Reactions were run on an ABI 9700 thermal cycler (Applied Biosystems) with the following cycling conditions: 98°C for 3 minutes, 20 cycles of 98°C for 30 s, 55°C for 30 s, 65°C for 1 minute with a final extension of 65°C for 10 minutes. Enriched reactions were purified using Agencourt AMPure XP beads (0.8× the reaction volume).

The hybridization reaction was prepared on ice in a 96-well plate with 100 ng enriched cDNA plus 1× hybridization buffer (50 mM HEPES, pH 7.3, USB; and 0.5 M NaCl, Ambion) in a final volume of 18 μl. The plate was incubated in a thermal cycler (ABI 9700) at 98°C for 10 minutes and 68°C for 4 h. A 68°C pre-heated mix of 2× DSN buffer (20 μl) and 2 μl DSN enzyme (Evrogen, Moscow, Russia) was added to each reaction (40 μl final volume) and incubated for another 25 minutes at 68°C. The reaction was stopped by adding 40 μl of the 2× DSN stop solution (10 mM EDTA) and purified with Agencourt AMPure XP beads (1.6× of the reaction volume). Samples were enriched with full-length Illumina PE1.0 and PE2.0 primers (1 μM each), 1× of AccuPrime PCR buffer I (10×), 0.5 U of AccuPrime Taq High Fidelity polymerase (5 U/μl; Invitrogen) in a final volume of 25 μl. Enriched reactions were purified using Agencourt AMPure XP beads (0.8× of the reaction volume) and sequenced.

### Illumina sequencing and data analysis pipeline

Libraries were sequenced on either Illumina GAII or Hi-Seq instruments. Sequencing mode (single or paired end) and read lengths for each experiment are available in Additional file 6. The raw reads of RNA-seq and DNA-seq data were processed using the Picard pipeline [41]. Briefly, the reads were aligned and assigned to the reference genomes using the program BWA [42], version 5.9, with parameters: -q 5 -l 32 -k 2 -t 4 -o 1. Sequence data for the PER mock community *(Prochlorococcus marinus *subsp. *pastoris *str. CCMP1986 (MED4), *Escherichia coli*, *K12 substr*. *MG1655*, *Rhodobacter sphaeroides 2.4.1*) were aligned to the respective genome sequences. BWA-aligned reads were then analyzed and assigned to individual genes according to the genome annotations provided by GenBank (*E. coli: *NC_000913.gff; *P. marinus*: NC_005072; *R. sphaeroides*: NC_007493.gff, NC_007494.gff, NC_007488.gff, NC_007489.gff, NC_007490.gff, NC_009007.gff and NC_009008.gff). The normalized read counts for each gene, RPKM, was calculated by 1,000 × (The sum of reads/Gene length) × (10^6^/Total mappable reads).

DNA-seq reads were aligned to a reference set of 649 selected bacterial genomes (Additional file 2). The initial list of the reference genomes and their annotations were downloaded from the Human Microbiome Project [6], which included 1,700+ organisms from GenBank and the draft genomes sequenced by the Human Microbiome Project. To reduce the misalignment and crosstalk between similar genomes, the reference genomes were aligned using all against all pairwise whole genome alignments in MUMMER [43] and clustered based on their MUMi values [44]. One representative from each genome cluster, sharing at minimum a MUMi value of 0.3, was selected for the final reference set. To further reduce the size of the reference set of 4 gigabases, BWA's upper limit, we removed genomes that had not been previously observed in healthy human gut microbiomes. Genomes to be eliminated were determined empirically from examination of whole genome shotgun sequencing data from hundreds of Human Microbiome Project samples, representing various body sites from 100 healthy individuals. To reduce the possibility of spurious alignment, the BWA-aligned reads were post-filtered at a minimum sequence identity of 97% to the best aligned reference genome. Since the human gut microbiome is often dominated by a handful of organisms, we chose the top 19 most abundant bacterial organisms observed in the meta-genomic data that had sufficient sequence coverage and depth to analyze the consequences of rRNA depletion from the same sample. Accession numbers for the reference sequences of these 649 species are listed in Additional file 2. Draft genomes lacking annotated rRNA genes were annotated in-house using the program RNAmmer [45]. The RPKM value for each gene was calculated as described above.

To measure strand specificity, we calculated the normalized abundance values for ORFs (RPKMO) as described [46]. Briefly, RPKMO values for regions corresponding to the sense and antisense strands of ORFs correspond to the number of reads aligning to these regions divided by the length of the region (in kb) and by the total number of reads aligning to the sense strand of all annotated ORFs in that sample (in millions). Annotations of protein-encoding genes were based on RefSeq NC_000913.gff, NC_005072.gff, NC_007493.gff, and NC_007494.gff for *E. coli*, *P. marinus*, and *R. sphaeroides *chromosome 1 and 2, respectively.

### Data availability

The sequencing data have been submitted to the Sequence Read Archive, and accession numbers are listed in Additional File 6.

## Abbreviations

CDS: coding DNA sequence; DSN: duplex-specific nuclease; ORF: open reading frame; PER pool: a pool (1:1:1 by mass) of total RNA extracted from *Prochlorococcus marinus*: *Escherichia coli*: and *Rhodobacter sphaeroides*; RIN: RNA integrity value; RPKM: reads per kilobases per million mappable reads; RPKMO: reads per kilobases per million mappable reads aligning to annotated open reading frames.

## Competing interests

The authors declare that they have no competing interests.

## Authors' contributions

DC and GG carried out research in the lab. KH, BJH, JL, DG and GG analyzed data. JZL advised on lab methods and data analyses. JI provided the human stool samples. AME and DW coordinated the transfer of stool samples to the Broad and participated in project discussions. BWB, CN and AG directed the project and coordinated the research. GG and AG wrote the paper. All authors have read and provided helpful comments for the manuscript, and they have approved the manuscript for publication.

## Supplementary Material

Additional file 1 **Figures S1 through S8 and legends**. Figure S1: linear correlation of gene expression profiles before and after different rRNA depletion methods. Figure S2: detrimental effect of high GC content on enrichment by low-c_0_t normalization using duplex specific nuclease (DSN). Figure S3: fragmentation profile of total RNA prior to Ribo-Zero treatment. Figure S4: PER CDS detection sensitivity before and after Ribo-Zero treatment of intact and fragmented RNA. Figure S5: antisense read densities for two technical replicates of strand-specific RNA seq of Ribo-zero treated PER RNA. Figure S6: representation of bacterial species in sequencing data sets from two human stool samples. Figure S7: RNA-seq data for *Prevotella copri*, *Bacteroides vulgatus*, *and Eubacterium rectale *in stool A. Figure S8: RNA-seq data for *Prevotella copri *and *Bacteroides vulgatus *in stool B. Click here for file

Additional file 2 **List of 649 reference genomes with GenBank accession numbers**. Click here for file

Additional file 3 **Total mapped reads to top 19 species in stool samples**. Click here for file

Additional file 4 **List of unannotated rRNAs in stool samples**. Click here for file

Additional file 5 **Sample recovery through the RNA-seq process assessed by the Agilent Bioanalyzer RNA 6000 Pico and DNA High Sensitivity Kits**. Click here for file

Additional file 6 **RNA-seq metrics and Sequence Read Archive accession numbers**. Click here for file
