# Supplementary material for: Efficient and robust RNA-seq process for cultured bacteria and complex community transcriptomes
Source: Genome Biol. 2012 Mar 28;13(3):r23. doi: 10.1186/gb-2012-13-3-r23 (PMC3439974; doi:10.1186/gb-2012-13-3-r23)
Supplement: Additional file 1 — Figures S1 through S8 and legends. Figure S1: linear correlation of gene expression profiles before and after different rRNA depletion methods. Figure S2: detrimental effect of high GC content on enrichment by low-c0t normalization using duplex specific nuclease (DSN). Figure S3: fragmentation profile of total RNA prior to Ribo-Zero treatment. Figure S4: PER CDS detection sensitivity before and after Ribo-Zero treatment of intact and fragmented RNA. Figure S5: antisense read densities for two technical replicates of strand-specific RNA seq of Ribo-zero treated PER RNA. Figure S6: representation of bacterial species in sequencing data sets from two human stool samples. Figure S7: RNA-seq data for Prevotella copri, Bacteroides vulgatus, and Eubacterium rectale in stool A. Figure S8: RNA-seq data for Prevotella copri and Bacteroides vulgatus in stool B. [file gb-2012-13-3-r23-S1.PDF]

Figure S1

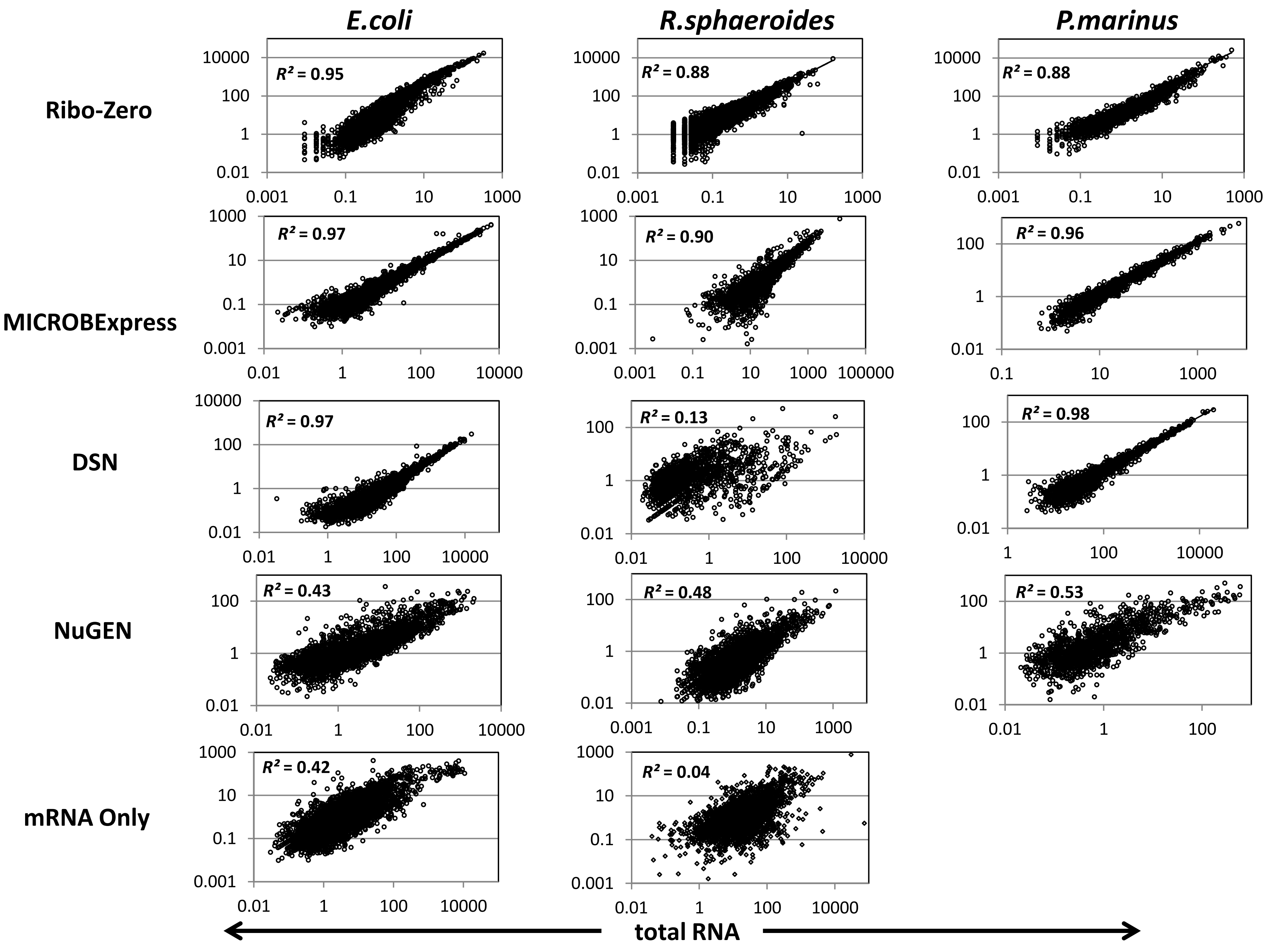
E.coli
R.sphaeroides
P.marinus
Ribo-Zero
MICROBExpress
DSN
NuGEN
mRNA Only
R² = 0.95
R² = 0.88
R² = 0.88
R² = 0.97
R² = 0.90
R² = 0.96
R² = 0.97
R² = 0.13
R² = 0.98
R² = 0.43
R² = 0.48
R² = 0.53
R² = 0.42
R² = 0.04
total RNA

Figure S2

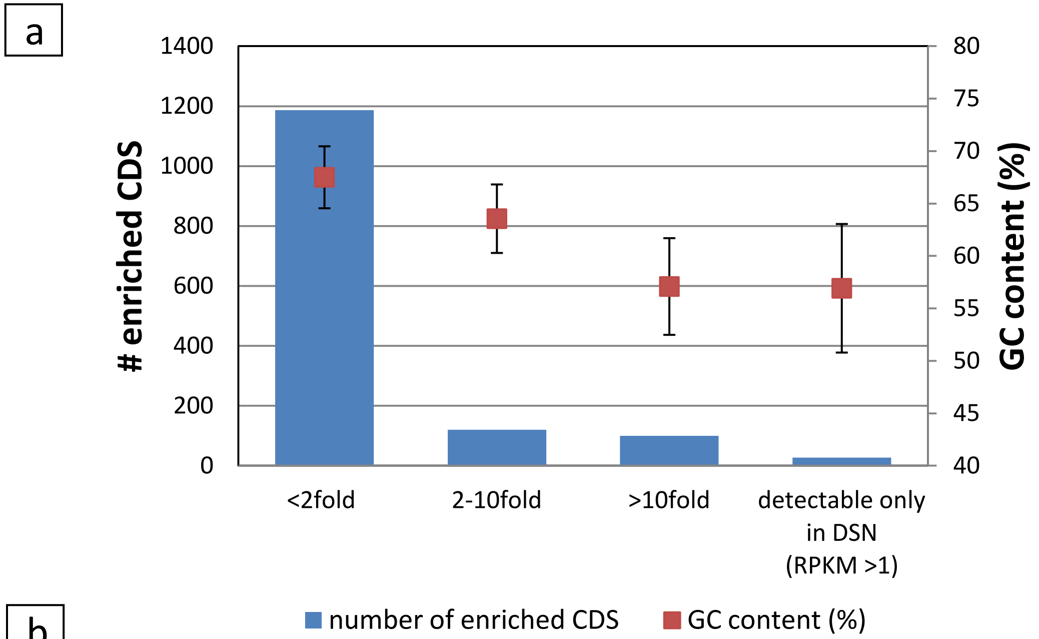
a
1400
1200
1000
800
600
400
200
0
enriched CDS
80
75
70
65
60
55
50
45
40
GC content (%)
<2fold
2-10fold
>10fold
detectable only in DSN (RPKM >1)
number of enriched CDS
GC content (%)

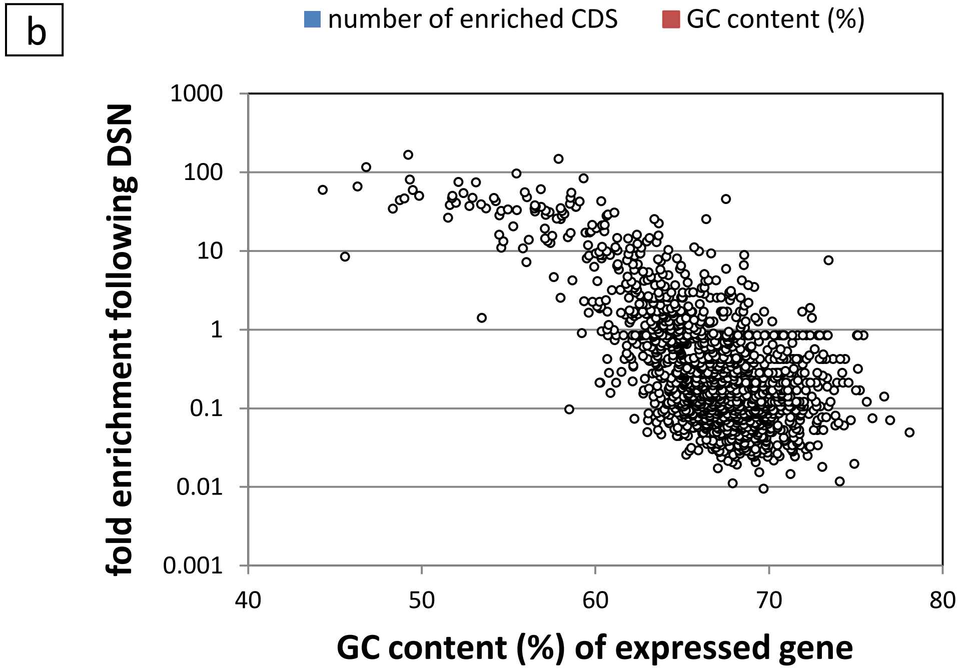
b
1000
100
10
1
0.1
0.01
0.001
fold enrichment following DSN
40
50
60
70
80
GC content (%) of expressed gene

Figure S3

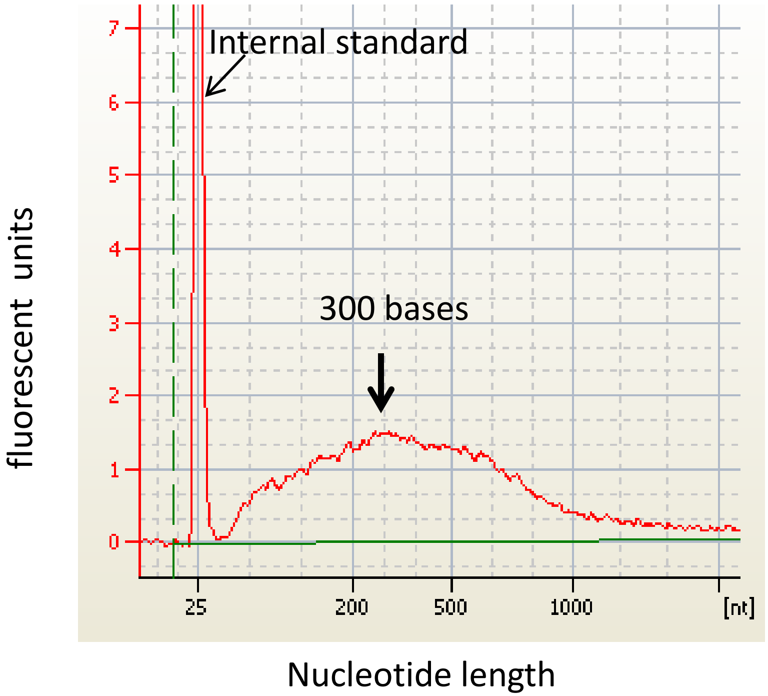
Internal standard
300 bases
fluorescent units
7
6
5
4
3
2
1
0
25
200
500
1000
[nt]
Nucleotide length

Figure S4

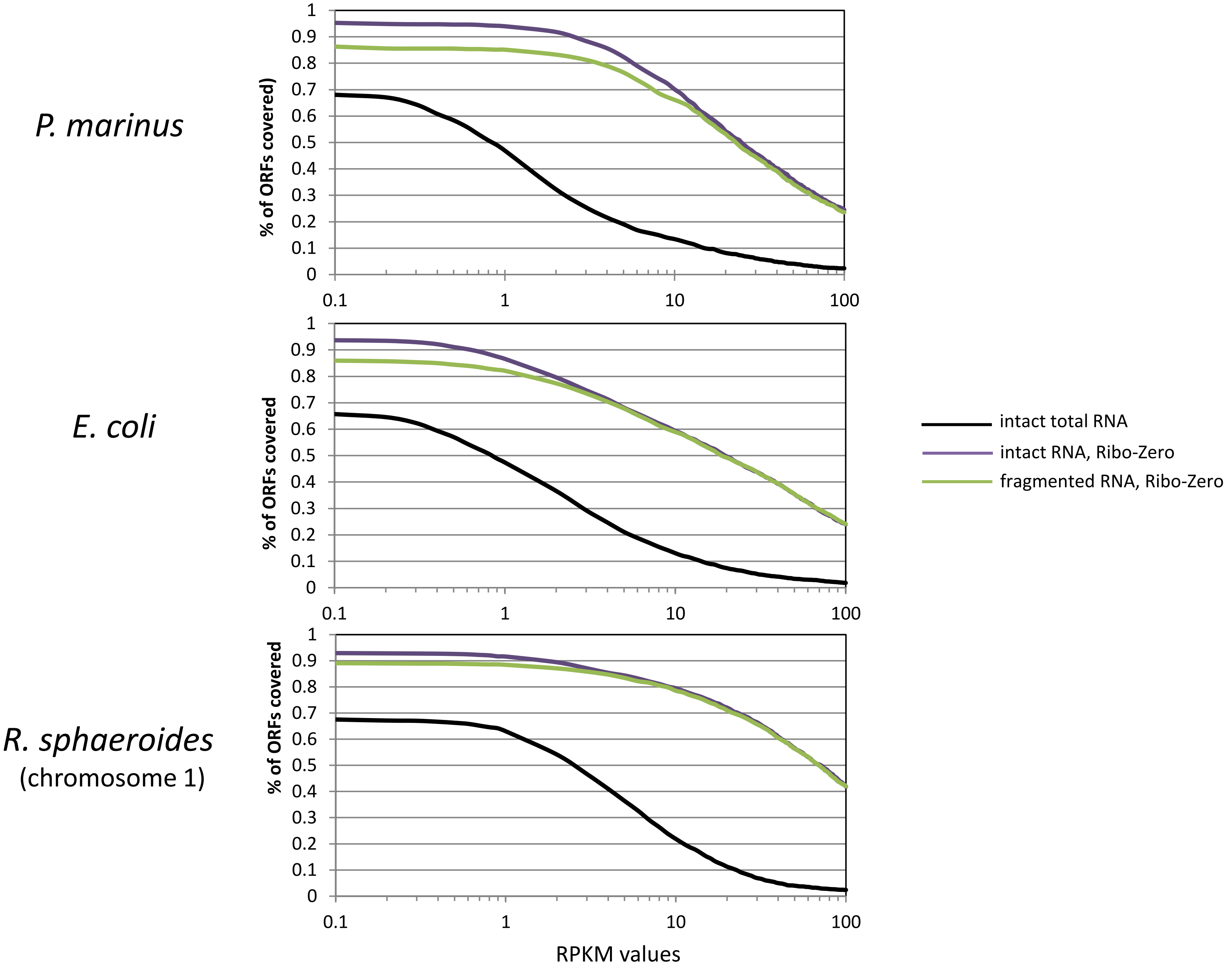
P. marinus
E. coli
R. sphaeroides
(chromosome 1)
% of ORFs covered)
% of ORFs covered
% of ORFs covered
1
0.9
0.8
0.7
0.6
0.5
0.4
0.3
0.2
0.1
0
0.1
1
10
100
RPKM values
intact total RNA
intact RNA, Ribo-Zero
fragmented RNA, Ribo-Zero

Figure S5

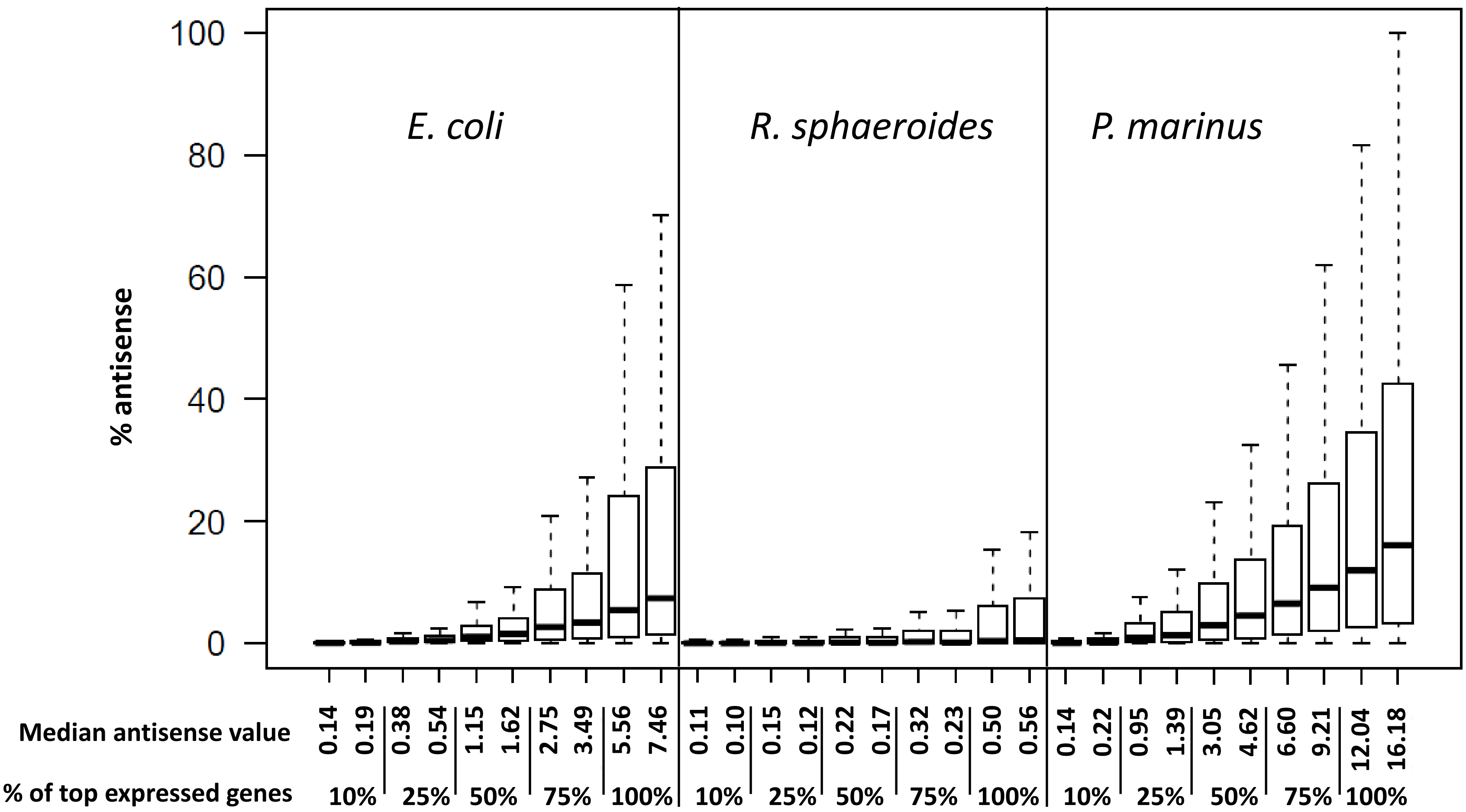

E. coli
R. sphaeroides
P. marinus
% antisense
100
80
60
40
20
0
Median antisense value
0.14
0.19
0.38
0.54
1.15
1.62
2.75
3.49
5.56
7.46
0.11
0.10
0.15
0.12
0.22
0.17
0.32
0.23
0.50
0.56
0.14
0.22
0.95
1.39
3.05
4.62
6.60
9.21
12.04
16.18
% of top expressed genes
10%
25%
50%
75%
100%
10%
25%
50%
75%
100%
10%
25%
50%
75%
100%

Figure S6

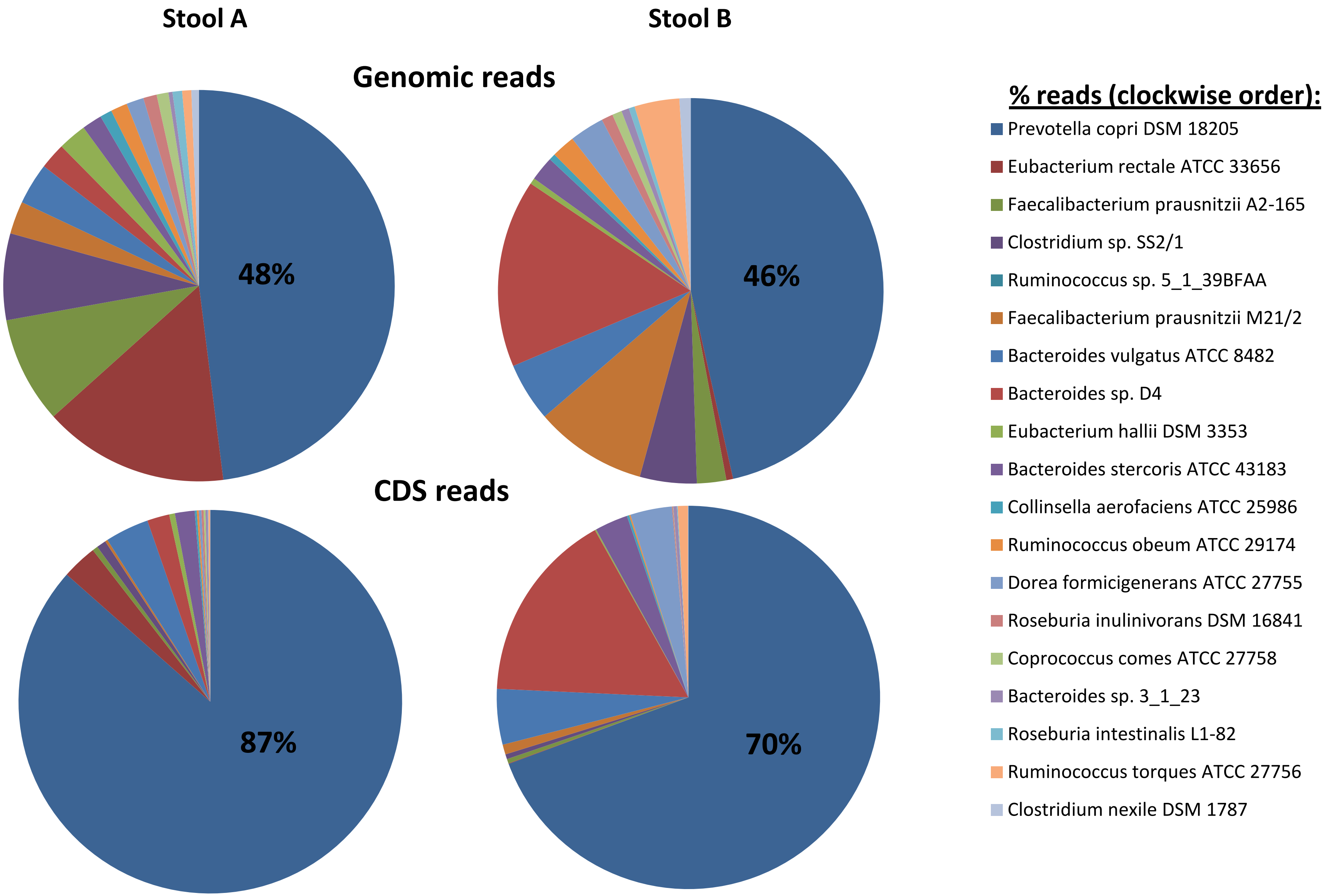

Stool A
Stool B
Genomic reads
48%
46%
CDS reads
87%
70%
% reads (clockwise order):
Prevotella copri DSM 18205
Eubacterium rectale ATCC 33656
Faecalibacterium prausnitzii A2-165
Clostridium sp. SS2/1
Ruminococcus sp. 5_1_39BFAA
Faecalibacterium prausnitzii M21/2
Bacteroides vulgatus ATCC 8482
Bacteroides sp. D4
Eubacterium hallii DSM 3353
Bacteroides stercoris ATCC 43183
Collinsella aerofaciens ATCC 25986
Ruminococcus obeum ATCC 29174
Dorea formicigenerans ATCC 27755
Roseburia inulinivorans DSM 16841
Coprococcus comes ATCC 27758
Bacteroides sp. 3_1_23
Roseburia intestinalis L1-82
Ruminococcus torques ATCC 27756
Clostridium nexile DSM 1787

Figure S7

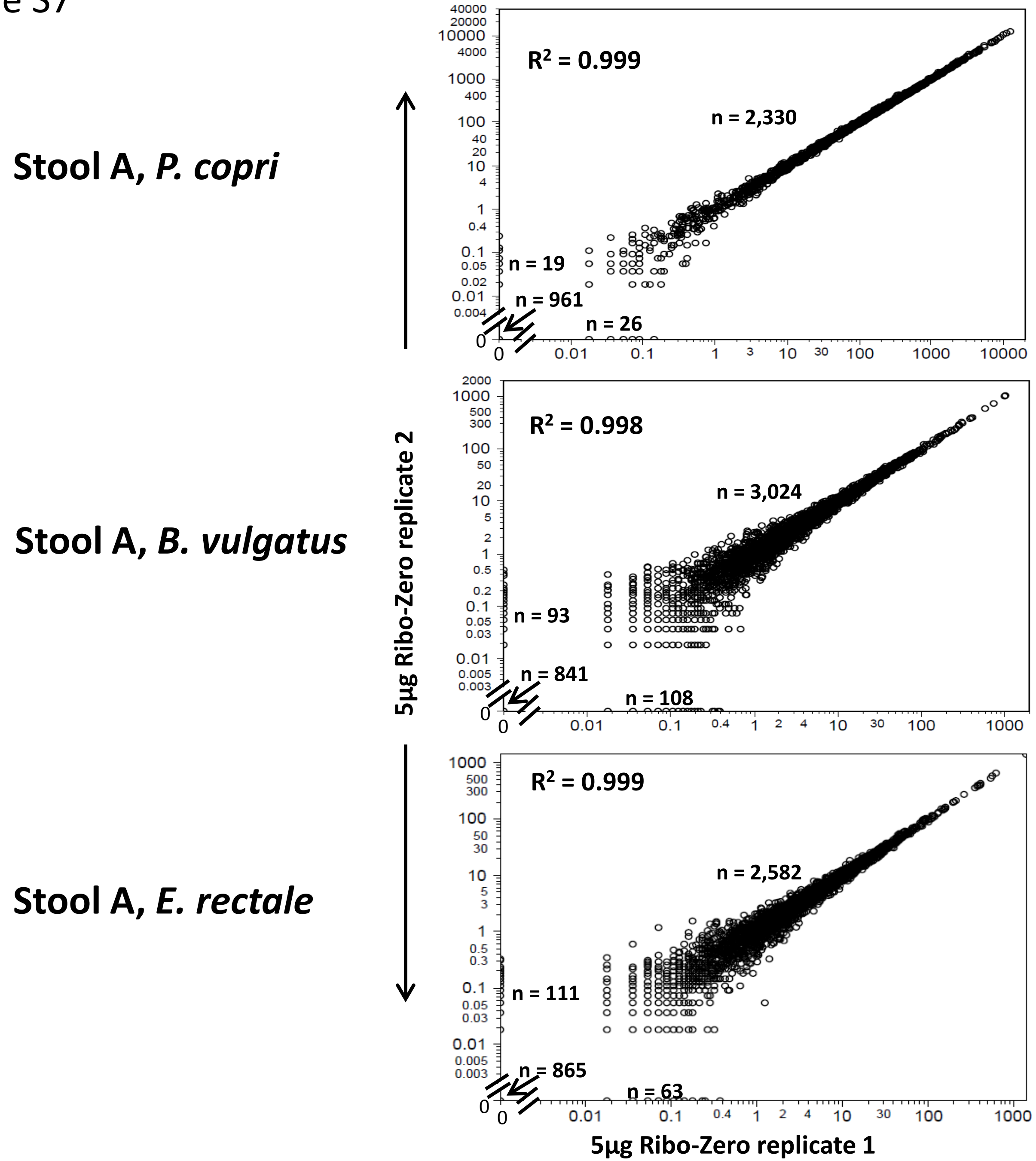
Stool A, P. copri
Stool A, B. vulgatus
Stool A, E. rectale
5µg Ribo-Zero replicate 2
5µg Ribo-Zero replicate 1
R² = 0.999
n = 2,330
n = 19
n = 961
n = 26
R² = 0.998
n = 3,024
n = 93
n = 841
n = 108
R² = 0.999
n = 2,582
n = 111
n = 865
n = 63

Figure S8

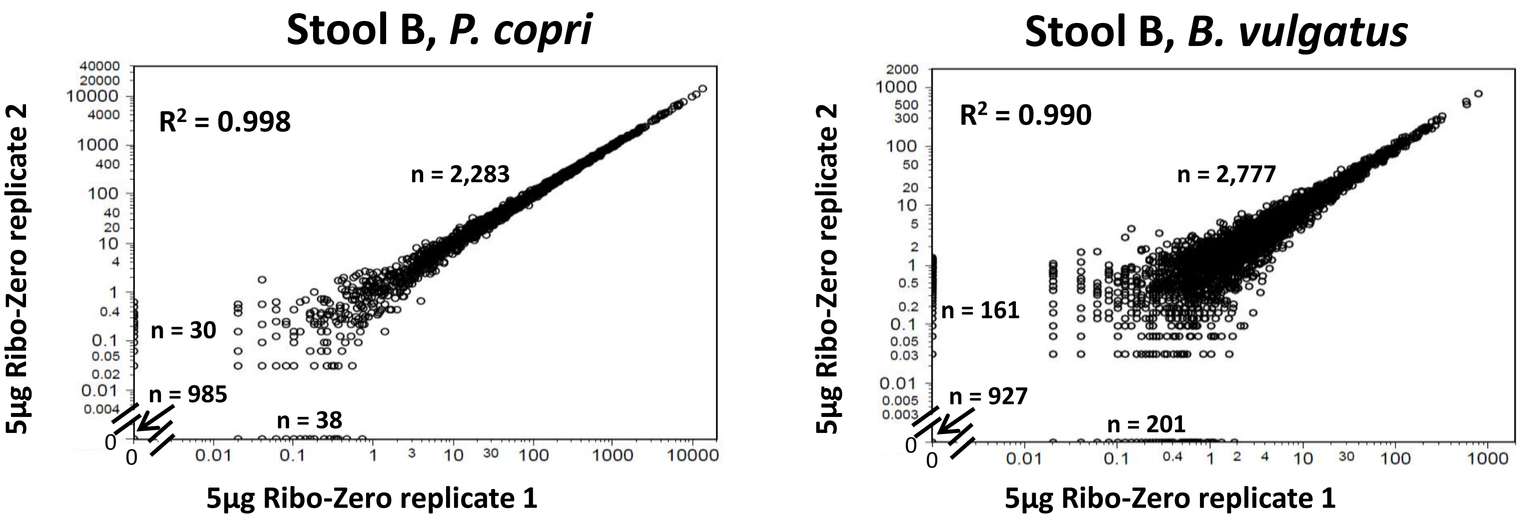

Stool B, *P. copri*
R² = 0.998
n = 2,283
n = 30
n = 985
n = 38
5µg Ribo-Zero replicate 2
5µg Ribo-Zero replicate 1
Stool B, *B. vulgatus*
R² = 0.990
n = 2,777
n = 161
n = 927
n = 201
5µg Ribo-Zero replicate 2
5µg Ribo-Zero replicate 1

Figure S1. Linear correlation of gene expression profiles before and after different rRNA depletion methods. Double-log scatter plots comparing RPKM values using five rRNA depletion methods (y-axes) with those obtained from undepleted total RNA (x-axes). The coefficients of determination (R^2) indicated for each plot is also shown in Figure 1b in the main text.

Figure S2. Detrimental effect of high GC content on enrichment by low-c_0t normalization using duplex specific nuclease (DSN). (a) Shown is the number of CDSs in *R. sphaeroides* that were enriched less than 2-fold, 2- to 10-fold, or >10-fold relative to the non-DSN control library. The bar on the right represents CDSs that were only detected in the DSN-treated library. The GC content (mean ± standard deviation) for each group is indicated. **(b)** Shown is the fold enrichment for 1407 *R. sphaeroides* CDSs plotted in a log scale over their respective GC content. The bulk of CDSs in *R. sphaeroides* were poorly enriched or even depleted relative to the control. CDSs with moderate GC (<60%) fared much better.

Figure S3. Fragmentation profile of total RNA prior to Ribo-Zero treatment. Shown is the profile of total RNA fragmented with Affymetrics fragmentation buffer (see Materials and methods) at 80°C for 4 minutes and analyzed on an Agilent Bioanalyzer RNA Nano chip.

Figure S4. PER CDS detection sensitivity before and after Ribo-Zero treatment of intact and fragmented RNA. Shown is the percentage of annotated CDSs with a normalized RNA-seq read density (RPKM) at or above the RPKM value indicated on the x-axis.

Figure S5. Antisense read densities for two technical replicates of strand-specific RNA seq of Ribo-zero treated PER RNA. The box plots represent the distribution of ratios (in %) of antisense (antisense/antisense + sense values) RPKMO values (see Materials and methods) for the top 10%, 25%, 50%, 75% and all expressed protein-coding genes for three bacterial species in replicate experiments. Horizontal lines, boxes and whiskers denote median, the two middle quartiles and 1.5 times the interquartile range, respectively.

Figure S6. Representation of bacterial species in sequencing data sets from two human stool samples. Shown is the distribution of genomic (DNA-seq) reads (top) and meta-transcriptomic (RNA-seq) CDS reads (bottom) among the 19 bacterial species indicated on the right. The percent of CDS reads in the pie charts correspond to the listed order of organisms in clockwise fashion.

Figure S7. **RNA-seq data for *Prevotella copri*, *Bacteroides vulgatus*, *and Eubacterium rectale* in stool A.** Shown are double-log scatter plots and the coefficients of determination (R^2) for RPKM values from technical replicates. Points on the axes represent CDSs without coverage in one of the two samples. The number of data points on the diagonal and on the axes is indicated.

Figure S8. RNA-seq data for *Prevotella copri* and *Bacteroides vulgatus* in stool B. Shown are double-log scatter plots and the coefficients of determination (R^2) for RPKM values from technical replicates. Points on the axes represent CDSs without coverage in one of the two samples. The number of data points on the diagonal and on the axes is indicated.
